# Illusory perception of visual patterns in pure noise is associated with COVID-19 conspiracy beliefs

**DOI:** 10.1177/20416695221144732

**Published:** 2023-01-18

**Authors:** Matthias Hartmann, Petra Müller

**Affiliations:** UniDistance Suisse, Switzerland; University of Bern, Switzerland

**Keywords:** illusory pattern perception, conspiracy beliefs, paranormal beliefs, COVID-19, cognitive biases

## Abstract

Just as perceptual heuristics can lead to visual illusions, cognitive heuristics can lead to biased judgements, such as “illusory pattern perception” (i.e., seeing patterns in unrelated events). Here we further investigated the common underlying mechanism behind irrational beliefs and illusory pattern perception in visual images. For trials in which no object was present in the noise, we found that the tendency to report seeing an object was positively correlated with the endorsement of both COVID-19 specific conspiracy theories and paranormal beliefs. The present results suggest that the cognitive bias to see meaningful connections in noise can have an impact on socio-political cognition as well as on perceptual decision making.

Telepathy, ghosts, astrology, or alternative treatments – humans sometimes believe in things that are not evidence-based and cannot be explained by scientific knowledge. The COVID-19 pandemic situation also has incited a flood of misinformation that often contradicts scientifically established knowledge, giving rise to conspiracy beliefs. Conspiracy beliefs are assumptions of secret plots by powerful evil groups who cover up information to suit their own interests (Douglas et al., 2017). Recent studies suggest that such beliefs undermine preventive behaviour, and are therefore a barrier in preventing the spread of SARS-CoV-2 (e.g., Hartmann & Müller, 2022; Pummerer et al., 2022). Given this relevance, the scientific interest in understanding the psychology of conspiracy beliefs has been exploded during the last 2 years.

Among others, a biased understanding of probability and causality has been suggested as a crucial mechanism behind the endorsement of conspiracy and paranormal beliefs, leading to illusory perception of meanings and patterns in unrelated events (e.g., Bressan, 2002; Matute et al., 2015). Interestingly, illusory pattern perception is not necessarily a purely cognitive bias but may also translate to perception. A few results suggest that “believers” are more likely to see meaningful patterns in Jackson Pollock paintings (van Prooijen et al., 2018), faces in natural images of trees (Riekki et al., 2013), or objects embedded in noise (Blackmore & Moore, 1994; van Elk, 2013). However, it remains unclear whether these results are based on individual differences in proneness/sensitivity to see hidden patterns, or a specific bias in perceiving illusory patterns in pure noise, and how different types of irrational beliefs (paranormal, conspiracy) are linked to such biases.

Here we further studied the possible common mechanisms of illusory pattern perception for paranormal and conspiracy beliefs, particularly focusing on COVID-19 specific beliefs. To this end, we presented 278 undergraduate students ten images from the modified Snowy Picture task (Whitson & Galinsky, 2008), with six of the pictures containing no objects and therefore allowing for illusory pattern recognition (see Figure 1 for example stimuli; the full set of stimuli can be asked from the authors). Participants were informed that there was an object hidden in some but not all of the stimuli, and for each stimulus they rated whether they perceived that there was an object (1  =  definitively no object, 6  =  definitively an object). Paranormal belief was measured by the Proneness to the Paranormal Scale (12 items), and COVID-19 conspiracy beliefs by five self-created items, such as ‘COVID-19 could have been stopped right at the start, but the large companies made a business out of keeping it going’ (see Hartmann & Müller, 2022 for full details of these measurements).

**Figure 1.**
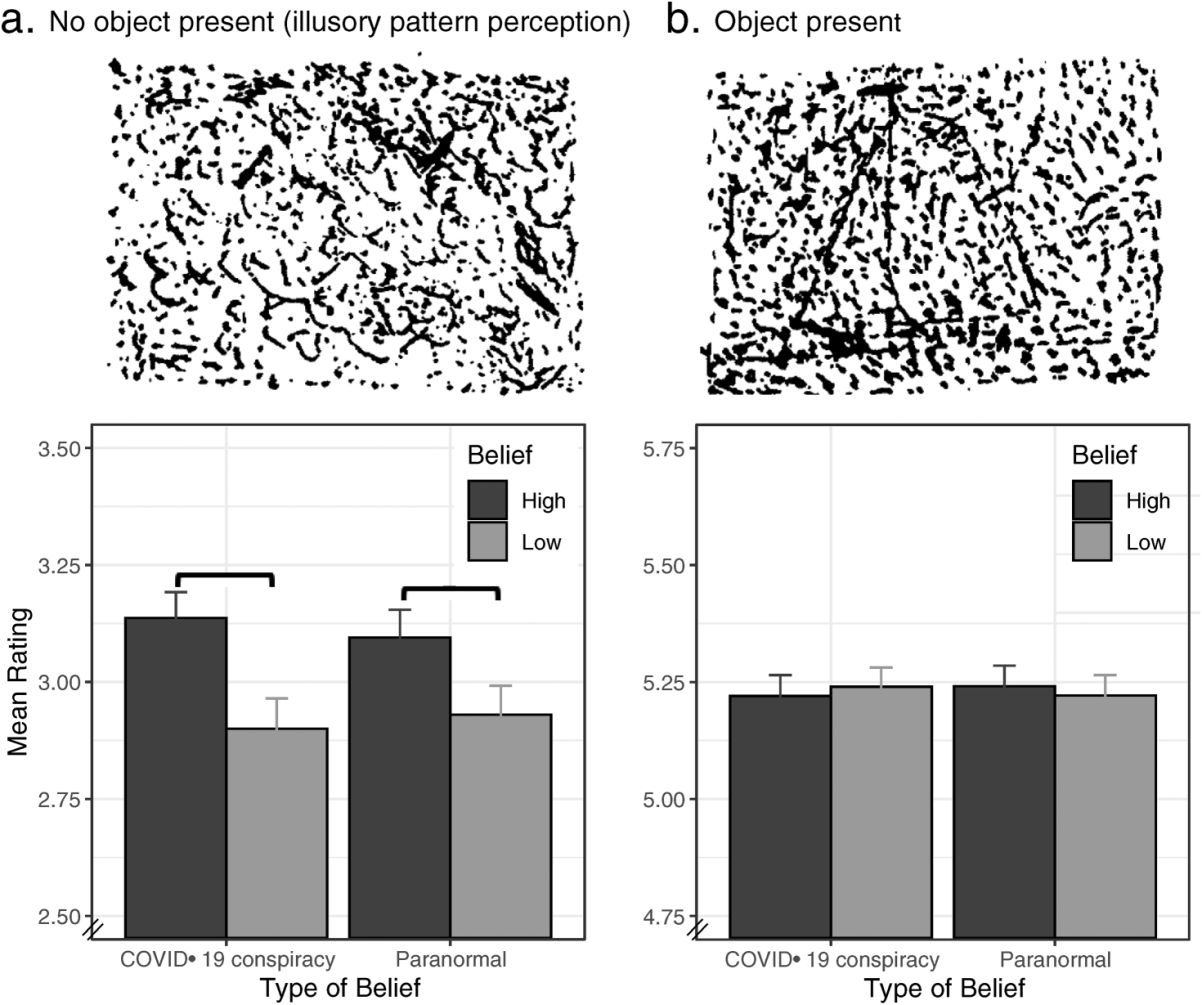
Example stimuli and mean ratings for trials with (a) no object present or (b) object present. Source of the stimuli: Whitson & Galinsky (2008).

Multivariate normality was violated, and we therefore used non-parametric analysis (Spearman's rho, Mann–Whitney *U*-test). COVID-19 conspiracy and paranormal beliefs were correlated (*r*  =  .38, *p* < .001). Most importantly, there was a highly significant positive correlation between COVID-19 conspiracy beliefs and ratings towards “object present” for trials in which there was no object (*r*  =  .21, *p* < .001). The same was found for paranormal beliefs, although less pronounced (*r*  =  .14, *p*  =  .024). No such correlations were found for trials in which objects were present (*p*s > .513). The results were also confirmed by dividing the groups into “low” and “high” believers based on median split (see Figure 1).

These results further suggest that illusory pattern perception is a common mechanism behind conspiracy and paranormal beliefs. The effect of belief was selective for situations in which there was no target present. This indicates that the endorsement of unwarranted beliefs is not associated with increased perceptual sensitivity (e.g., Blackmore & Moore, 1994) but rather with an increased likelihood to report seeing something when there actually is nothing (Krummenacher et al., 2010; Riekki et al., 2013). Regarding conspiracy beliefs, it is known that epistemic mistrust (government, science) and a biased processing of (mis)information play key roles (Pierre, 2020). The present results suggest that the cognitive bias to see meaningful connections in noise can have an impact on socio-political cognition as well as on perceptual decision making.

As a limitation, only a small set of stimuli was used in this study, and the signal-to-noise-ratio of stimuli was not manipulated systematically. Future studies should extend these findings using a more sophisticated signal detection theory approach, and also further explore the role of specific personal traits and needs that are associated with both illusory pattern perception and irrational beliefs (e.g., Darwin et al., 2011; Gligorić et al., 2021).
